# Detection of *Mycoplasma suis* in pre-suckling piglets indicates a vertical transmission

**DOI:** 10.1186/s12917-019-2001-y

**Published:** 2019-07-19

**Authors:** Julia Stadler, Stephan Willi, Mathias Ritzmann, Matthias Eddicks, Julia Ade, Katharina Hoelzle, Ludwig E. Hoelzle

**Affiliations:** 1 Clinic for Swine, Centre for Clinical Veterinary Medicine, LMU Munich, Sonnenstrasse 16, 85764 Oberschleissheim, Germany; 2 Institute of Animal Science, University of Hohenheim, Garbenstrasse 30, 70593 Stuttgart, Germany

**Keywords:** *Mycoplasma suis*, Pre-suckling piglets, Vertical transmission

## Abstract

**Background:**

Transmission of *Mycoplasma (M.) suis* mainly occurs via iatrogenic or zootechnical manipulations or due to ranking fights. Other transmission routes including ingestion of secretes/excretes; blood-sucking arthropods and intra-uterine transmission have thought to play an epidemiological role without being experimentally proven. To investigate a vertical transmission of *M. suis* under field conditions blood samples from pre-suckling piglets and their corresponding dam were examined for *M. suis* by quantitative polymerase chain reaction *(*qPCR) in 21 farms in Southern Germany*.*

**Results:**

*A* total of 14.35% of the 474 blood samples from pre-suckling piglets reacted qPCR positive. Additionally, *M. suis* was detected in 65 (31.25%) of the 208 sows at farrowing*.* On farm level, 16 (76.2%) of the 21 farms had at least one *M. suis* positive animal. *M. suis* positive farms had an average of 0.41 more stillborn piglets per litter than *M. suis* negative farms (*p* = 0.007).

**Conclusion:**

The present study provides further insights into *M. suis* infection dynamics as it is the first detection of *M. suis* in piglets immediately after birth prior to colostrum intake and the first large scale investigation of *M. suis* in sows at farrowing.

## Background

*M. suis*, the causative agent of infectious anemia in pigs (IAP), is an important pathogen in modern intense pig production worldwide [1–5]. *M. suis* affects all age classes of pigs. In piglets, acute IAP manifests as life-threatening hemolytic anemia, general ill thrift, and hypoglycemia which could lead to acute death [2]. In sows, acute *M. suis* infections may cause sudden death due to hypoglycemic coma but also milder acute forms of the disease including decreased fertility, increased return to estrus and dysgalactia have been reported [1, 6–8]. However, main *e*conomic losses associated with *M. suis* infections in all age classes are related to chronic IAP with mild anemia, reduced growth rate, poor reproductive performance, increased antibiotic use and a higher susceptibility to secondary infections of the respiratory and enteric system [5].

*M. suis* belongs to the highly specialized group of hemotrophic mycoplasmas with special unique features including cell tropism to erythrocytes and endothelial cells, a reduced genome and a high metabolic host adaption [5, 9–12]. All previous efforts to cultivate *M. suis* in vitro have been unsuccessful so far, although a kind of maintenance after nanotransformation can be obtained in a cell free culture system [13].

Due to the inability to cultivate hemotrophic mycoplasmas, reliable prevalence data for *M. suis* are rare and restricted to the post-PCR era. Moreover, it is supposed that *M. suis* infections have been underdiagnosed due to the low sensitivity and specificity of former diagnostic methods like microscopic examination of blood smears coupled with whole blood which is rarely included in routine diagnostic submission, being the preferred sample type [6]. Nowadays, diagnostic of *M. suis* infection is mainly based on PCR techniques or serological examinations of relevant animal groups (whole cell ELISA or recombinant ELISAs) [4, 14–16]. In applying qPCR methods *M. suis* prevalence of 13.9 and 10.0% has been determined for weaned piglets and wild boars in Germany, respectively [3, 17] and of 18.2% in sows in Brazil [18]. Nevertheless, one key question that remains unknown is the introduction of *M. suis* into swine herds and the on-farm transmission between pigs. It is proven that transmission of infected blood occurs via iatrogenic or zootechnical procedures (vaccinations, contaminated needles, fixation procedures) or lacerations due to ranking fights within animal groups [19, 20]. Moreover, other transmission routes including ingestion of secretes and excretes, blood-sucking arthropods and intra-uterine transmission have thought to play an epidemiological role without however being experimentally proven [1, 7, 19, 21]. The aim of the present study was to determine the occurrence of vertical *M. suis* transmission from dams to their offspring under field conditions. Therefore, blood samples of sows at farrowing and their pre-suckling piglets were investigated by means of an *M. suis*-specific qPCR. Furthermore, the impact of *M. suis* infection on the piglet producing farms was evaluated by correlating the qPCR results to hematological findings as well as reproductive performance data.

## Results

### *M. suis* detection in sows at farrowing and pre-suckling piglets

In all farms no clinical signs of *M. suis* infections were obvious at the time of investigation. In 16 (76.2%) out of the 21 investigated farms *M. suis* was detected in at least one sow, in the remaining five farms (23.8%) all sows were qPCR-negative. On individual animal level, 31.25% (65 out of 208) of the sows were positive for *M. suis*. The number of *M. suis* positive sows within herds varied between 1 and 10 animals with a mean number of 3.05 (SD ± 2.99) positive sows per farm.

To investigate the vertical transmission of *M. suis* from sows to their offspring, all samples from pre-suckling piglets (*n* = 474) from the 16 *M. suis* positive farms with 65 *M. suis* positive sows and 94 *M. suis* negative sows were investigated for the presence of *M. suis*. Overall 68 (14.35%) of 474 pre-suckling piglets reacted qPCR positive. The 68 *M. suis* positive piglets originated from 47 litters. Table 1 gives an overview on the number of positive piglets according to the *M. suis* status of the sow in the 16 *M. suis* positive farms. Fifty (73.5%) piglets were born from 32 *M. suis* positive sows and 18 (26.5%) piglets from 15 sows that showed a PCR negative result at the time of sampling. Piglets born from a *M. suis* positive dam were significantly more often positive than piglets born from a *M. suis* negative dam (*p* < 0.001, OR: 3.8, 95% CI: 1.8, 8.5). Quantification of bacterial loads revealed a mean *M. suis* blood load of 3.15 × 10^7^ *M. suis*/mL in sows (range.: 2.04 *M. suis*/mL to 1.94 × 10^9^ *M. suis*/mL blood) and of 5.09 × 10^7^ *M. suis*/mL blood in piglets, respectively (range: 1.02 *M. suis*/mL to 3.46 × 10^9^ *M. suis/mL* blood). Bacterial blood loads of sows were associated with bacterial blood loads of piglets (*p*<0.001). No significant difference was observed between birth-weight of *M. suis* positive and negative pigs. The median birth weight was 1.35 kg for *M. suis* positive piglets and 1.40 kg for *M. suis* negative piglets. Furthermore, no significant association was found between the gender and the *M. suis* status of the piglet. Among the 65 *M. suis* positive sows, 7 sows originated from parity group 1, 31 sows from parity group 2 and 27 sows were in parity group 3. However, the parity of the sow was neither associated with the *M. suis* status of the sows nor with the number of positive piglets per sow.

**Table 1:** Number of *M. suis* positive piglets per sows according to the *M. suis* status of the sow in the 16 *M. suis* positive farms

Number of positive piglets per sow	*M. suis* positive sows (*n* = 65)	*M. suis* negative sows (*n* = 94)
0	33	79
1	16	13
2	14	1
3	2	1

### Immunoblot analysis

Sera from qPCR negative sows with *M. suis* positive piglets (*n* = 15) and from negative sows with *M. suis* negative piglets (*n* = 15) were investigated for the presence of *M. suis*-specific antibodies. Sera from all sows (100%) with *M. suis* positive piglets reacted positive in the *M. suis*-immunoblot, whereas only 1 of the 15 sows without *M. suis* positive piglets (6.66%) revealed a positive serological result. Detailed reaction patterns of the immunoblot-positive sows are given in Table 2.

**Table 2:** Reaction patterns of all immunoblot positive sows

*M. suis* specific antigen^a^	qPCR negative sows with qPCR positive piglets (*n* = 15)															qPCR negative sow with qPCR negative piglets (*n* = 1)
	1	2	3	4	5	6	7	8	9	10	11	12	13	14	15	16^c^
p 83	+ ^b^															
p 73																
p 70																
p 61																
p 57		+									+		+	+		
p 45	+			+	+	+	+					+				+
p 40		+	+	+	+	+	+	+			+	+	+	+	+	
p 33	+	+	+					+	+	+					+	+
p 31																

^a^*M. suis* antigen purified from experimentally infected animals at high bacteremia

^b^distinct visible reactions with *M. suis* antigen and absent in the control antigen purified from the blood of *M. suis* negative sows

^c^reaction pattern of the one immunoblot-positive sow of the control group. All other sera from the control group sows (*n* = 14) showed no reaction with any *M. suis* specific antigen and were thus not included in the table

### Hematological findings and correlation between qPCR and hematological parameters

In the group of sows, no significant differences in hematological parameters (erythrocyte, leucocyte, PCV, hemoglobin and thrombocyte) could be observed between *M. suis* positive and *M. suis* negative animals. Additionally, no correlation between *M. suis* blood load of sows and hematological parameters was found. However, erythrocyte (*p*<0.001), PCV (*p* = 0.02) and hemoglobin count (*p* = 0.002) were negatively associated with the parity of the sow. *M. suis* positive piglets had significantly higher leucocyte counts (median: 6.49. g/l) than *M. suis* negative piglets (median: 5.60 g/l) (*p*<0.001). Additionally, birth weight was positively associated with leucocyte count (*p* = 0.001). The remaining hematological parameters (erythrocyte, PCV, hemoglobin and thrombocytes) did not differ significantly between *M. suis* positive and *M. suis* negative piglets. However, *M. suis* blood load of piglets was negatively correlated with erythrocyte count (*r* = − 0.243, *p* = 0.046) and positively correlated with leucocyte count (*r* = 0.548, *p*<0.001).

### Reproductive parameters of *M. suis* positive and negative farms

**Fig. 1:**
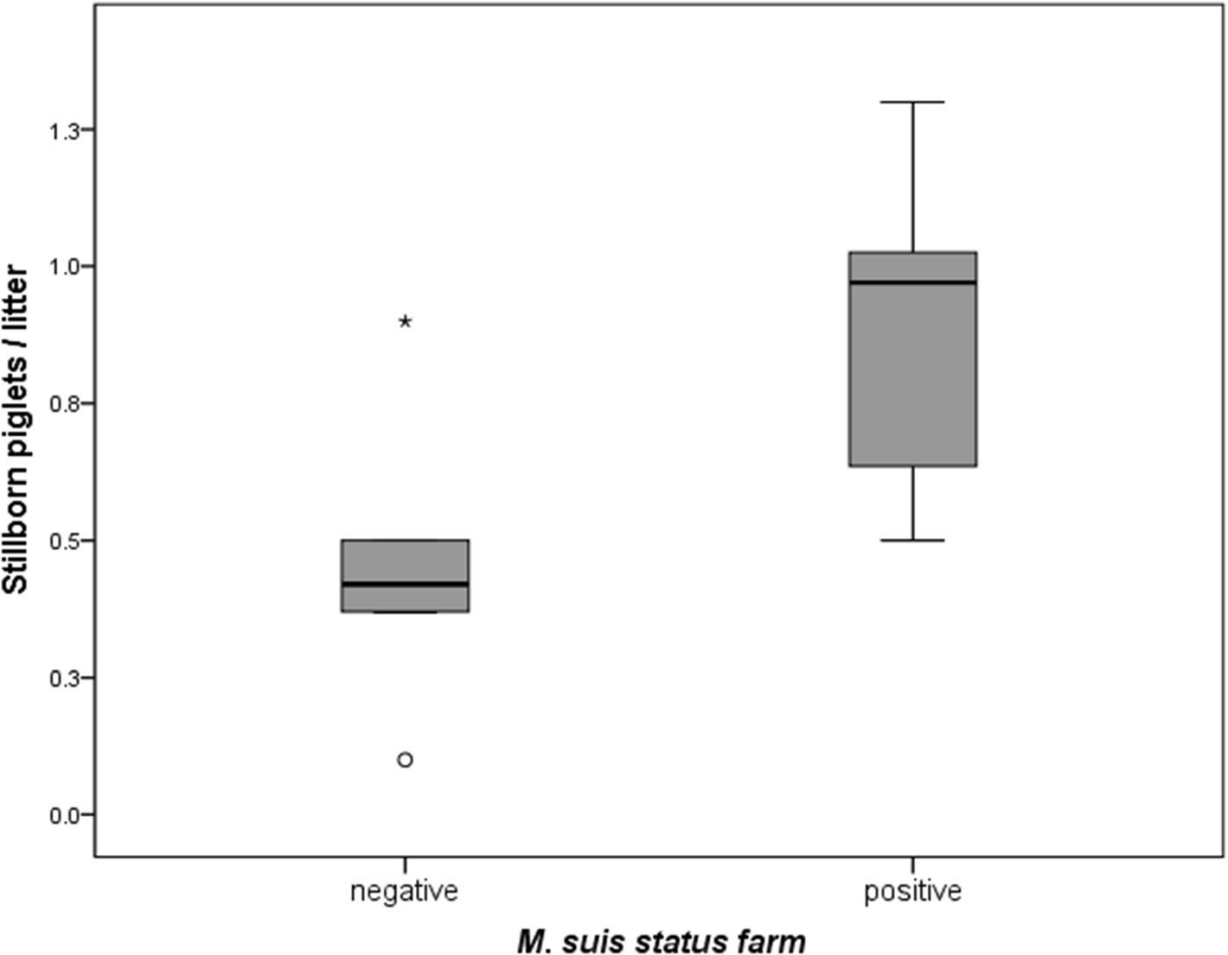
Mean number of stillborn piglets per litter in *M. suis* positive and *M. suis* negative farms. Outliers are shown as O, extreme outliers as *

Regarding reproductive parameters, sows on *M. suis* positive farms had significantly more stillborn piglets per litter (an average of 0.41 more) compared to *M. suis* negative farms (*p* = 0.007) (Fig. 1). Other evaluated reproductive parameters (i.e. live born piglets/sow, weaned piglets/sow, return to estrus rate) did not differ significantly between *M. suis* positive and negative farms.

## Discussion

This study reports the evidence of *M. suis* in sows at farrowing and their corresponding piglets without obvious clinical signs of infection at the time of investigation. However, *M. suis* is able to persist in asymptomatic carrier animals and reoccurrence of the disease can be provoked by immunosuppressive events (e.g. stress, transport, other infectious agents). Additionally, subclinically infected carrier animals can be regarded as the major reservoir of *M. suis* and play an important role in the epidemiology of infections [22]. The study involved a total of 208 sows from 21 piglet producing farms and 474 piglets from 16 *M. suis* positive farms. We found that 14.35% of the pre-suckling blood samples of newborn piglets from *M. suis* positive farms were qPCR positive indicating that the vertical route might play an important role in the transmission of *M. suis* within herds. Nearly 50% (32 out of 65) of the *M. suis* positive sows have born at least one *M. suis* positive piglet. This finding was unexpected because it is generally accepted that transmission of *M. suis* mainly occurs horizontally including an iatrogenic blood transfer due to contaminated instruments, small skin lesions due to hierarchy fights within animal groups or a transmission due to shedding via secretes and excretes [4, 19, 20]. So far, vertical transmission was discussed to play an epidemiological role without being experimentally proven*.* Only one previous study of Henderson et al. [7] suggested a vertical transmission as *M. suis* was detected in piglets shortly after birth but after colostrum intake and intensive contact between piglets and dam. Vertical transmission of hemotrophic mycoplasma has only been described so far in cattle [23, 24]. To the best of our knowledge, this is the first detailed study investigating *M. suis* infection in sows at farrowing and the potential vertical transmission of *M. suis* to piglets.

There are two main possibilities for the piglets to get infected: intra-uterine or due to blood or secret contact during birth passage (e.g. vaginal lesions or vaginal secret). The latter was supported by the detection of vaginal *M. suis* shedding in experimentally infected pigs [19]. However, according to the high *M. suis* mean blood load of 5.09 × 10^7^ detected in piglets immediately after expulsion, amplification of the pathogen due to uptake of secretions or contamination with sow blood during parturition seems unlikely. Results from a previous experimental trial revealed lower mean *M. suis* blood loads of 1.35 × 10^3^ and 5.36 × 10^5^ on day 2 post infection in non splenectomized and splenectomized nursery pigs, respectively [2].

Basically, the *M. suis* blood loads found in the present study seem to be very high for both, the sows and the piglets. However, they are comparable to the loads found in 164 *M. suis* positive feeder pigs in Germany with a mean load of 7.62 × 10^7^ *M. suis*/mL blood [3]. Interestingly, 18 *M. suis* positive piglets (26.5%) were born from PCR negative sows. However, we could detect *M. suis* specific antibodies in the sera of all *M. suis* negative sows with *M. suis* positive offspring indicating a prior exposure to *M. suis* or even a chronic *M. suis* infection with a bacterial blood load below the PCR detection limit of 10 *M. suis* per reaction [25]. Such an intermittent detection of *M. suis* has also been described previously [2]. Due to the high percentage of *M. suis* positive piglets derived from PCR positive sows it seems likely that the fetal outcome is dependent on the *M. suis* status of the sow. Future experimental studies are certainly needed to gain deeper insights into the mechanism by which *M. suis* transmits from the dam to her fetuses and to elucidate the pathogenesis of embryonal/fetal *M. suis* infection.

Twenty-one farms with 208 sows of different parities were included in the present study. The high detection rate of 76.2% *M. suis* positive piglet producing farms and 31.25% *M. suis* positive sows, indicates that subclinical *M. suis*-infection is widespread in clinically healthy sows. Comparable studies are rare. There is only one PCR based study in healthy sows from Brazil reporting 18.2% *M. suis* positive animals [18]. In feeder pigs 13.9% of the animals and 40.3% of the farms were *M. suis* PCR-positive in Germany [3]. Other studies on sows investigating *M. suis*-specific antibodies revealed inconsistent results including 59% seropositive sows in Portugal [26] as well as 39.2 to 40.6% seropositive replacement gilts and 47.0 to 48.2% seropositive multiparous sows in China, respectively [27, 28]. Various factors could be responsible for different prevalences, especially the chosen diagnostic method (PCR or serology) and the study design. One disadvantage of the PCR methodology used in the present study might be that, in contrast to pathogen isolation, non-viable bacteria can be detected. However, due to the lack of in vitro cultivation systems for *M. suis* and other hemotrophic mycoplasmas, PCR is currently the most sensitive detection method. Prevalence data can also be biased by other factors i.e. varying epidemiologic situation in different countries or the selected age group as *M. suis* prevalence is thought to increase with age [18, 26–28]. However, in contrast to Song et al. [28] who reported a higher prevalence in multiparous sows compared to gilts no parity dependent differences could be observed in our study. Additionally, the sampling point at farrowing might have influenced the detection rate of *M. suis* in our study as stress or immunosuppression is thought to increase the susceptibility for *M. suis* [4, 29].

Several clinical syndromes have been associated with *M. suis* infections in sows including acute and chronic anemia, pyrexia, anorexia, hypoglycemia, icterus but also reproductive disorders with decreased fertility, increased return to estrus, decreased number of live born and weaned piglets, abortion, mummies, and dysgalactia [1, 6]. In this study, clinical signs were not obvious at the time of investigation. However, the different courses of *M. suis* infections (acute, chronic or latent) are mainly dependent on endogenous or exogenous stress factors [22]. As clinical examination of sows was only performed once at the day of farrowing and reproductive performance was only assessed at farm level and not from individual animals further studies focusing on individual reproductive performance of *M. suis* positive sows are certainly needed. Additionally, no evidence linking *M. suis* infections to anemia in sows has been found. This lack of correlation is in accordance with Guimaraes et al. [18] who couldn’t determine significant differences in hematological parameters between infected and not infected sows. One possible explanation for non-observed differences in hematological parameters between positive and negative sows in the present study might be that alteration of hematological parameters in *M. suis* positive sows was overlaid by other factors eg. parity of the sow.

Interestingly, in the present study *M. suis* positive newborn piglets had significantly higher leucocyte counts than *M. suis* negative piglets and leucocyte counts were positively correlated with *M. suis* blood loads. Additionally, the negative correlation between *M. suis* blood loads and erythrocyte counts is in accordance with Ritzmann et al. [3] who showed that bacterial loads are significantly correlated with severity of anemia. The absence of obvious clinical signs in newborn piglets might also be attributed to the fact that in compliance with the German welfare legislation only clinically healthy piglets were included in the study. Further studies should focus on the clinical outcome of piglets born *M. suis* positive, particularly after stressing conditions (e.g. weaning).

In the present study *M. suis* positive farms had a significantly higher number of stillborn piglets compared to *M. suis* negative farms. Other reproductive parameters including return to estrus and number of live born/weaned piglets did not differ significantly between *M. suis* positive and negative farms. Reproductive performance of sows can be influenced by several infectious and non-infectious co-factors. Samples of the present study were also investigated for porcine circovirus type 2 resulting in a low prevalence in farrowing sows (1%) and no detection in suckling piglets as published by Eddicks et al. [30]. To investigate other potential coinfections that might influence the outcome of the present study samples were also examined for PRRSV indicating no link between PRRSV and *M. suis* infection (data not published). However, the higher number of stillborn piglets in *M. suis* positive farms must be interpreted cautiously as other infectious as well as non-infectious agents influencing reproductive parameter were not evaluated within the scope of this study.

## Conclusion

In the present study the detection of *M. suis* in pre-suckling piglets indicates for the first time a potential vertical transmission of this pathogen. The high detection rate of *M. suis* in clinically healthy sows suggests that sows play a role in within herd transmission. Therefore, the present study increases our knowledge on *M. suis* infection dynamics and transmission, thus improving adequate and effective intervention strategies.

## Methods

### Sample and data collection

Out of a pool of 36 voluntary participating piglet producing farms 21 farms, regardless of their *M. suis* status, were randomly selected stratified by the density of piglet producing farms in Bavaria, Germany. The number of farms to sample was determined based on expert knowledge, average number of farms sampled in literature and considering financial and logistic constraints. The investigation of 200 sows allows the estimation of prevalence with accuracy up to ±7%. Accordingly, 10 sows per farm were sampled in the 21 selected farms. This kind of two stage cluster sampling is a frequently used sampling method that ensures high practicability and validity of observed data at the same time. On the other hand, the examination of 40 animals per farm allows the detection of a 10% minimal prevalence of *M. suis* DNA on a farm with a 98% confidence level. Therefore, 30 piglets per farm were sampled (three piglets per sow). The size of the farms varied between 100 and 840 sows with an average farm size of 294 sows. EDTA-anticoagulated blood samples and serum samples were collected from 9 or 10 sows at the time of farrowing from each farm (*n* = 208). Additionally, EDTA-anticoagulated blood samples were collected from three piglets of each sow (*n* = 622) before colostrum uptake as described by Eddicks et al. [30]. In accordance with the German animal welfare law only clinically healthy pre-suckling piglets were included in this investigation and subsequently piglets were raised as conventional pigs. To ensure that blood sampling of piglets was performed prior to the first suckling of the piglets the whole farrowing period was supervised by the investigators and piglets were sampled immediately after expulsion (< 30 s. between birth and sampling). Analysis of hematological parameters and qPCR analysis was performed from EDTA-anticoagulated blood samples. After analysis of hematological parameters EDTA-anticoagulated blood samples were stored at − 80 °C until further processing. Serum samples of sows were examined for *M. suis* specific antibodies by immunoblot analysis. Animal based data were collected including birth weigth and sex of each piglet. The parity group of each sow was recorded according to the following scheme: parity group 1 (gilts), group 2 (2nd-4th parity) and group 3 (≥5th parity) and sows were examined for obvious clinical signs of *M. suis* infection (anorexia, depression, anemia, icterus, pyrexia) at the day of farrowing. Additionally, reproductive parameters (return to estrus rate, live born piglets/sow/litter, stillborn piglets/sow/litter, weaned piglets/sow/year) were assessed at farm level on each farm. All procedures were performed in accordance with the German animal welfare law using a protocol officially approved by the appropriate authority (reference number: 55.2–154–2532.2-16-13).

### DNA extraction

Two hundred microlitre of EDTA-anticoagulated blood samples were pre-treated as described previously [3, 31]. Afterwards, bacterial DNA was extracted from the samples using the GenElute™ Bacterial Genomic DNA Kit (Sigma-Aldrich, Steinheim, Germany) according to the manufacturer’s instructions. One PBS control was included in each DNA extraction run (1 control for 10 samples) to monitor for cross-contamination. DNA was stored at − 20 °C until use.

### Quantitative SYBR green real time PCR

*M. suis* DNA was detected and quantified with the StepOne™ System (Applied Biosystems®) and the primers targeting the *M. suis msg*1: *msg*1-Fw 5'-ACAACTAATGCACTAGCTCCTATC-3' and *msg*1-Rv 5'-GCTCCTGTAGTTGTAGGAATAATTGA). Real-time PCR (qPCR) was performed by means of Fast SYBR® Green Master Mix (ThermoFisher Scientific) with 0.5 μM of each primer. The SYBR green PCR protocol comprised 95 °C for 10 min followed by 40 cycles of 95 °C for 15 s and 60 °C for 30 s. After each PCR a melting curve analysis was performed with melting temperatures of 76.0 ± 0.1 °C were considered as positive. Specificity testing of the SYBR green real time PCR assay was performed using DNA samples from the following bacteria: *M. hyorhinis, M. hyopneumoniae, M. wenyonii*, '*Candidatus* M. haemobos', *M. haemofelis, Salmonella* Typhimurium, *Escherichia coli, Pasteurella multocida, Streptococcus suis*. Determination of the lower detection limit as well as quantification of *M. suis* blood loads in positive pigs was performed as described previously [25]. The detection limit of the SYBR green real time PCR assay was found to be 10 *M. suis* per PCR reaction.

### Hematological and biochemical blood analyses

Hematological parameters including erythrocyte, hemoglobin, leucocyte and thrombocyte counts as well as packed cell volume (PCV), mean corpuscular volume (MCV), mean

corpuscular hemoglobin (MCH) and mean corpuscular hemoglobin concentration

(MCHC) were determined using the Vet Scil ABC tool (Scil Animal Care Company GmbH, Viernheim, Germany).

### Immunoblot analysis

Serum samples were examined for antibodies against *M. suis* by immunoblot analysis as described by Hoelzle et al. [15]*.* Briefly, antigen preparations derived from *M. suis*-infected pigs and negative pigs were separated on sodium dodecyl sulfate-polyacrylamide gels according to their molecular weight and transferred to nitrocellulose membranes by standard methods. The immunoblots were probed with field sera from the sows diluted 1:100, horseradish peroxidase-labeled goat anti-pig IgG (Sigma-Aldrich), and with 4-chloro-1-naphthol as the chromogenic reagent. Immunoreactive protein bands were sized with reference to molecular size marker lanes (Page Ruler prestained Protein ladder, Thermofisher Scientific). Samples were considered positive if at least one of the three major immunogenic proteins p40, p45, and p70 was detected [15].

### Statistical evaluation

Data were compiled and analyzed with Microsoft Office Excel 2013 and the statistic software IBM SPSS, Statistics 22.0 (IBM Corporation, USA) and RStudio (Version 1.1.453 with R Version 3.4.4.). A farm was considered positive if at least one animal tested positive. Data were tested for normal distribution using Kolmogorow Smirnow test. Multivariable analysis were carried out using linear mixed model (hematological parameters of piglets and sows, birth weight of piglets, bacterial load of sow and piglets) and logistic model (status sow, piglet, gender of the piglets, parity of the sows) accounting for repeated measures (farm and sow effect). Pearson correlation coefficient was used to determine a correlation between bacterial loads of sows respectively piglets and hematological parameters. The significance level of all statistical evaluations was 5% with a 95% confidence interval.

## Data Availability

The datasets used and/or analysed during the current study are available from the corresponding author on reasonable request.

## Abbreviations

*IAP*: *Infectious anemia in pigs*
*M. suis*: *Mycoplasma suis*
MCH: Mean corpuscular hemoglobin
MCHC: Mean corpuscular hemoglobin concentration
MCV: Mean corpuscular volume
PCV: Packed cell volume
qPCR: Quantitative polymerase chain reaction
